# Data driven pathway analysis and forecast of global warming and sea level rise

**DOI:** 10.1038/s41598-023-30789-4

**Published:** 2023-04-04

**Authors:** Jiecheng Song, Guanchao Tong, Jiayou Chao, Jean Chung, Minghua Zhang, Wuyin Lin, Tao Zhang, Peter M. Bentler, Wei Zhu

**Affiliations:** 1 Department of Applied Mathematics and Statistics, State University of New York at Stony Brook, Stony Brook, NY 11794-3600 USA; 2 Duke University, 2080 Duke University Road, Durham, NC 27708 USA; 3 School of Marine and Atmospheric Sciences, State University of New York at Stony Brook, Stony Brook, NY 11794-5000 USA; 4 Environmental and Climate Sciences Department, Brookhaven National Laboratory, Upton, NY 11973-5000 USA; 5 Department of Statistics, University of California, Los Angeles, Los Angeles, CA 90095-1554 USA

**Keywords:** Climate sciences, Climate change, Climate-change impacts, Projection and prediction

## Abstract

Climate change is a critical issue of our time, and its causes, pathways, and forecasts remain a topic of broader discussion. In this paper, we present a novel data driven pathway analysis framework to identify the key processes behind mean global temperature and sea level rise, and to forecast the magnitude of their increase from the present to 2100. Based on historical data and dynamic statistical modeling alone, we have established the causal pathways that connect increasing greenhouse gas emissions to increasing global mean temperature and sea level, with its intermediate links encompassing humidity, sea ice coverage, and glacier mass, but not for sunspot numbers. Our results indicate that if no action is taken to curb anthropogenic greenhouse gas emissions, the global average temperature would rise to an estimated 3.28 °C (2.46–4.10 °C) above its pre-industrial level while the global sea level would be an estimated 573 mm (474–671 mm) above its 2021 mean by 2100. However, if countries adhere to the greenhouse gas emission regulations outlined in the 2021 United Nations Conference on Climate Change (COP26), the rise in global temperature would lessen to an average increase of 1.88 °C (1.43–2.33 °C) above its pre-industrial level, albeit still higher than the targeted 1.5 °C, while the sea level increase would reduce to 449 mm (389–509 mm) above its 2021 mean by 2100.

## Introduction

As of 2021, the global mean surface temperature had already risen by 1.21 °C over its pre-industrial level (1850–1900 average)^1^, while the global mean sea level had increased by 82 mm compared to its 1986–2005 average^2^. Global warming and sea level rise are two critical indicators of climate change. The record breaking summer of 2022 has sent an urgent and harsh reminder for action and resolution, with temperatures exceeding 40 °C in many parts of Europe and the USA^3^. Identifying the causal pathways leading to this change based on historical, open-box data and models that clarify the often complicated physical processes involved will provide more transparency on the role and significance of greenhouse gas emissions in driving climate change. In this work, we use the unified structural equation modeling (uSEM) approach developed by our research group^4^ to identify the climate change pathways leading to increased global mean surface temperature (GMST) and global mean sea level (GMSL) based on yearly historical data. Backtesting is performed to validate the pathway models. The confirmed pathway models are then used to forecast future GMST and GMSL based on (1) the unrestricted scenario assuming the global community will make no significant actions to contain the anthropogenic greenhouse gas emissions, (2) the COP26 scenario of restricted anthropogenic carbon dioxide (CO_2_) and methane (CH_4_) emissions, and (3) the SSP (Shared Socioeconomic Pathways) scenarios of greenhouse gases concentration projection. We have also modeled the link between global sea level to regional sea level and illustrated the impact of regional sea level rise on coastal metropolises such as New York City, USA, and Osaka, Japan, by 2050 and 2100.

Many previous studies have endeavored to establish causal relations and predictions for global mean surface temperature (GMST), and global mean sea level (GMSL)^5–8^. Traditionally physical models utilizing complex process models of the general circulation of the planet’s atmosphere and ocean have been employed to generate global climate change predictions, with strict boundary and starting conditions^9–24^. These models can be used to comprehend the fundamental dynamics of the physical components of the natural climate phenomena, derive global temporal and spatial changes, and make predictions based on the future greenhouse gases emissions^25^. Differences between simulations and observations are often expected, with significant differences usually caused by numerical approximations, nonlinear behaviors, unresolved small-scale processes, and variability in data^26–28^. Therefore, the projections of the physical models can range widely^29^.

In the last few decades, data-driven methods utilizing time series analysis and machine learning methods have been increasingly adopted to forecast global surface temperature or sea level^5–7,30–41^ and local climate^28,42–48^. Data-driven models attempt to obtain evidence of externally driven climate change while minimizing the use of complex climate models. The advantages are usually simpler models and lower computational burdens. These models can be typically divided into two categories: cointegration approaches^49^ and regression approaches^50^. Cointegration is a statistical technique used to find the equilibrium connection between two or more non-stationary time series over the long run. The cointegration method assumes that non-stationary time series have a long-term relationship. These models, however, are not designed to capture the complex inter-relationships among climate variables^46^. Unlike physical models, data-based models often suffer from data inconsistency and absence more severely. Machine learning methods, including deep learning, have been applied to examine global climate change recently^28,30,31,41,44^. However, machine learning methods are often regarded as black boxes, with the model’s underlying dynamics unseen to the users. In addition, machine learning methods require large amount of training data which are often unattainable in climate change studies.

In 2021, we published the first work^7^ on pathway analysis of global warming and sea level rise utilizing monthly historical data. In this paper, we have drastically extended the climate change network analysis by adopting much longer yearly data, representing the effect of greenhouse gas emissions in a more comprehensive manner, adding humidity and sunspot activities, and improving the pathway identification process by using both the system-wise and the equation-wise variable selection methods. Importantly, for the forecasting of GMST and GMSL based on the confirmed pathway models, we have focused on two scenarios: (1) without any restriction on anthropogenic greenhouse gas emission, and (2) with restriction on anthropogenic greenhouse gas emission as outlined in the 2021 United Nations Conference on Climate Change (COP26). To make our analysis more comparable, we also included SSP scenarios in this work. Finally, we have expanded the regional sea level projections beyond the US coastal line to include other global regions heavily affected by climate change.

## Results

### Path discovery and analysis of global mean surface temperature (GMST) and global mean sea level (GMSL)

**Figure 1:**
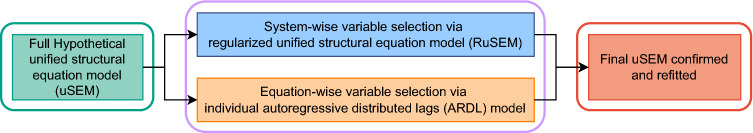
Flow chart of unified structural equation model (uSEM) pathway identification process using two variable selection methods, the system-wise regularized unified structural equation modeling (RuSEM) approach, and the equation-wise stepwise variable selection method using each individual uSEM equation, which is also an autoregressive distributed lags (ARDL) model. The fully hypothesized uSEM model and the final confirmed uSEM pathway model are depicted in Fig. 2a,b, respectively. For this work, the two variable selection methods identified a common final pathway model as shown in Fig. 2b below.

**Figure 2:**
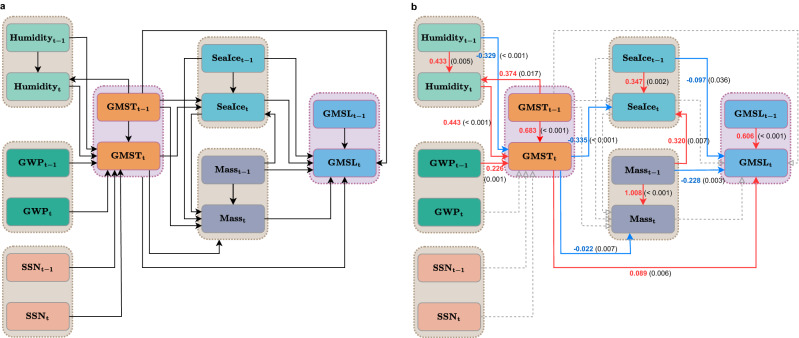
(**a**) Full hypothesized unified structural equation model (uSEM), and (**b**) the confirmed uSEM with significant pathways selected in unison by two variable selection methods—the system-wise regularized uSEM (RuSEM) approach and the equation-wise stepwise variable selection based on each ARDL model. Climate Factors included (from left top to right bottom) are: Global Specific Humidity (Humidity), Global Warming Potential (GWP), Sunspot Number (SSN), Global Mean Surface Temperature with sea ice area measured by air above sea ice (GMST), Arctic Sea Ice August Extent (Sea Ice), Glaciers and Ice Sheets Mass Balance (Mass), and Global Mean Sea Level (GMSL). In (**a**), the full hypothesized path model with all conceivable directed paths not contradictory to common sense as depicted by the black arrows. In (**b**), the final path model identified, significant positive or negative pathways are labeled with red or blue arrows with the corresponding path coefficients and p-values (in parentheses, 1-sided) labeled, while grey dashed arrows represent insignificant pathways at the significant level of 0.05 (1-sided).

To analyze the causal pathways between GMST, GMSL, and other climate factors, we have considered 9 variables: (1) Global Mean Sea Level (GMSL), (2) Glaciers and Ice Sheets Mass Balance (Mass), (3) Arctic Sea Ice August Extent (SeaIce), (4) Global Mean Surface Temperature with the sea ice area temperature measured by air (GMST), (5) Global Specific Humidity (Humidity), (6–8) Greenhouse Gases: CO_2_, CH_4_, and N_2_O (which are represented jointly by their total global warming potential (GWP) to avoid multi-collinearity), and (9) Sunspot Number (SSN), a measurement of solar activity. We started with a full hypothetical unified structural equation model (uSEM)^4^ that includes all conceivable paths by only excluding those contradictory to common sense (Fig. 2a). The uSEM approach can incorporate both the contemporaneous and the longitudinal pathways, with the uSEM equation system consisting of a set of autoregressive distributed lags (ARDL) models. Subsequently, two variable selection methods were applied to select significant pathways based on historical data (Fig. 1).

First, a system-wise variable selection for the entire uSEM equation system was conducted using the adaptive LASSO (least absolute shrinkage and selection operator) in the regularized unified structural equation modeling (RuSEM) approach^51^. Secondly, an equation-wise variable selection was performed on each ARDL model of the uSEM equation system using the backward stepwise variable selection based on the Akaike information criterion (AIC). In our analysis, these two variable selection methods yielded the same selected model, confirming the robustness of the climate change pathways identified via uSEM. The identified uSEM system with significant paths was only refitted to obtain the estimated path coefficients (red for positive and blue for negative path coefficient values) and the corresponding p-values in parentheses (Fig. 2b). Backtesting using both the one-step forecast as well as the multi-step forecast methods was performed to further validate the identified climate change pathways. This uSEM path model is entirely data-driven, open-box with explicit equations, and with the minimum use of existing scientific knowledge for an intuitive and independent inference of the status and the future of our climate system in addition to the sophisticated physics-driven models which are often represented as a black-box to the laymen.

The data confirmed uSEM pathway as shown in Fig. 2b indicates that increases in greenhouse gas emission, in terms of the increased Global Warming Potential (GWP), would significantly increase the Global Mean Surface Temperature (GMST), while the Sunspot Number (SSN) has no significant impact on GMST. Humidity and GMST have a positive mutual interactive relationship. Furthermore, the increase in GMST would significantly decrease the Sea Ice coverage (SeaIce) and the Glaciers and Ice Sheets Mass Balance (Mass), which would in turn significantly increase the Global Mean Sea Level (GMSL). The melting glaciers also have a direct hand in the melting (or reduced formation) of sea ice, possibly due to the decreased ice sheet coverage leading to less reflective ice surfaces, or a decreased albedo, thus leading to a greater absorption (rather than reflection) of solar radiation that creates a positive feedback loop for increasing temperatures and the increased melting of ice sheets and sea ice. Sea ice is critical in the regulation of temperature, albedo, and ocean circulation, therefore a decrease in sea ice would lead to significant consequences for global climate. Notably, it has a significant role in driving the global ocean conveyor belt by sinking salty, denser water that forms underneath its surface, which circulates along the ocean floor and drives the flow of the warmer, less dense water on the surface^52^. An increase in GMST would also promote a higher GMSL directly besides its indirect role through Sea Ice decrease and Glacier melting.

Notably, the data-driven model yielded pathways highly consistent with recent scientific findings. GWP, for instance, has a positive impact on GMST, in agreement with the scientific verdict of the greenhouse effect^53,54^. The positive mutual relationship between Humidity and GMST is consistent with the theory of the water vapor greenhouse effect and the water cycle theory, which states that warmer air contains more water vapor^55^, thus increasing evaporation and reducing condensation. It is sensible that higher GMST results in melting of the glaciers, ice sheets, and sea ice due to warmer temperature^56,57^. The negative impact from Mass or Sea Ice to GMSL can be explained similarly^58^. Besides, the melting of Sea Ice may have contributed to GMSL rise indirectly through reduction in solar reflection and ocean thermal expansion. The positive impact from GMST to GMSL is consistent with the generally held view that the GMSL increase is partially driven by the thermal expansion of the oceans^58,59^. In short, the data driven pathway analysis results align well with the principles of climate science. Detailed information about the data and methods, as well as detailed descriptions of the consistency between this data-driven approach and those based on physics, are shown in the Method section.

### Forecast of global mean surface temperature (GMST) and global mean sea level (GMSL)

The most recent United Nations Conference on Climate Change (COP26) was held in 2021 in Glasgow, Scotland, gathering almost 200 nations to discuss the critical issue of climate change and global strategy. To combat climate change, nations are urged to establish ambitious emission reduction plans. The conference participants agreed to a new climate deal, the Glasgow Climate Pact, aiming to keep GMST within 1.5 °C above its pre-industrial levels. According to the Glasgow Climate Conference^60^, it is essential that the anthropogenic CO_2_ emissions should decrease by 45% by 2030 relative to its 2010 level, and the anthropogenic CH_4_ emissions should decrease by 30% by 2030 relative to its 2020 level in order to achieve this goal. However, several studies^61–63^ have since questioned the sufficiency of the COP26 guidelines. Our research aims to answer the following questions by using pure data-driven models based on historic data: (1) What will the GMST and GMSL be by 2100 if the anthropogenic greenhouse gas emission is not controlled per COP26 regulations (namely, the unrestricted scenario)? (2) What will the GMST and GMSL be by 2100 if COP26 regulations are followed through (namely, the COP26 scenario)?

As shown in Fig. 2b, the GWP is solely incorporated in our path model as a predictor. In other words, no ARDL model in the uSEM equation system includes GWP as a response variable. For forecasting, we can model the trend of GWP changes from both anthropogenic and natural causes by fitting classic autoregressive integrated moving average (ARIMA) time series models, to historical data. The best fitting time series model for the historical data is an ARIMA(1,1,1) model. For forecasting under the COP26 scenario, our model calculates the future values of anthropogenic GWP of CO_2_ and CH_4_ using the COP26 guidelines assuming a constant rate of emission reduction while modeling the unrestricted component of GWP using the ARIMA(1,1,1) model. Permafrost can also contribute to future greenhouse gas emissions^64^. To incorporate permafrost’s effect in the forecast model, the forecast of CO_2_ and CH_4_ emissions from the Arctic permafrost under medium and low scenarios according^64^ are added to the forecast of GWP under the unrestricted scenario and COP26 restricted emission scenario correspondingly. Details about permafrost greenhouse gas emission can be found in the Supplementary Materials.

**Figure 3:**
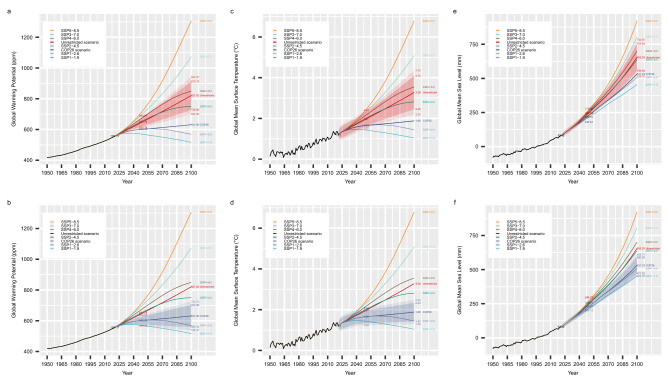
Data driven forecast of Global Warming Potential (GWP) (**a**,**b**), Global Mean Surface Temperature (GMST) (**c**,**d**), and Global Mean Sea Level (GMSL) (**e**,**f**) under the unrestricted scenario (red), the COP26 scenario (grey), SSP5-8.5 (orange), SSP3-7.0 (light-green), SSP4-6.0 (brown), SSP2-4.5 (dark-green), SSP1-2.6 (light-grey) and SSP1-1.9 (light-blue) from now till 2100, the uncertainties (forecast interval) under the unrestricted and the COP26 scenarios were shown by the red and grey shaded area. For the unrestricted and the COP26 forecast, values of the expected means and the 95% and 99% forecast intervals are shown for 2050 and 2100 respectively. Historical data before 2022 are shown in black. The modeling and forecast of GWP was based on an estimated ARIMA(1,1,1) model using historical data for the unrestricted scenario and integrating the proposed restriction for the COP26 scenario (with greenhouse gas emitted by Arctic permafrost added). The estimation and forecast of GMST and GMSL are based on the confirmed uSEM model as shown in Fig. 2b.

Our uSEM pathway model (Fig. 2b) predicts that under the unrestricted scenario, GMST will increase to 1.97 °C above its pre-industrial level (or 0.76 °C above its 2021 level) by 2050, and 3.28 °C above its pre-industrial level (or 2.07 °C above its 2021 level) by 2100 (Fig. 3c). Using the 20-year mean level from 1986 to 2005 as the baseline, GMSL will increase to 246.72 mm (or 164.30 mm above its 2021 level) by 2050, and 655.25 mm (or 572.83 mm above its 2021 level) by 2100 (Fig. 3e). In contrast, under the COP26 scenario, GMST will increase to 1.66 °C above its pre-industrial level (or 0.45 °C above its 2021 level) by 2050, and 1.88 °C above its pre-industrial level (or 0.67 °C above its 2021 level) by 2100 (Fig. 3d). The GMSL will increase to 229.67 mm (or 147.25 mm above its 2021 level) by 2050, and 531.23 mm (or 448.81 mm above its 2021 level) by 2100 (Fig. 3f). Table 1 summarizes these results, with the inclusion of confidence intervals. This study and the projections indicate that the mandates set by COP26 will have a substantial influence on climate change mitigation. However, our results indicate that the proposed plans may fall slightly short of keeping the global temperature rise below 1.5 °C over its pre-industrial level, as we forecast an average warming of 1.88 °C based on COP26 guidelines. Therefore, the COP26 guidelines are proven to be insufficient in keeping warming within 1.5 °C to curb the effects of severe climate change. To put in perspective, we have also included several Shared Socioeconomic Pathways (SSP) scenarios for the greenhouse emissions^65^. We use future concentrations for CO_2_, CH_4_, and N_2_O under the SSP scenarios^66,67^ to forecast the GMST and GMSL. The forecasts for the GMST and GMSL under different SSP scenarios by using our pathway model are also shown in Fig. 3. Under SSP 5-8.5 scenario, which is the most extreme scenario, GMST will increase to 2.43 °C above its pre-industrial (or 1.22 °C above its 2021 level) by 2050, and 6.79 °C above its pre-industrial (or 5.58 °C above its 2021 level) level by 2100. GMSL will increase to 271.93 mm (or 189.51 mm above its 2021 level) by 2050, and 921.64 mm (or 839.22 mm above its 2021 level) by 2100. As shown in Fig. 3, our projection of the unrestricted scenario is closest to the SSP 2-4.5 scenario, while our projection of COP26 scenario is closest to the SSP 1-2.6 scenario.

**Table 1:** Predicted means and forecast intervals (95% and 99%) of GMST rise and GMSL rise by 2050 and by 2100 under unrestricted or COP26 restrictions on greenhouse gas emissions; and the predicted means of GMST rise and GMSL rise by 2050 and by 2100 under SSP1-1.9, SSP1-2.6, SSP2-4.5, SSP3-7.0, SSP4-6.0 and SSP5-8.5 scenarios. The baseline for GMST is the pre-industrial (1850–1900 average) level, while the baseline for GMSL is the 20-year mean value of GMSL from 1986 to 2005. The units of GMST are °C and the units of GMSL are mm.

	Year	Unrestricted scenario			COP26 scenario		
		Mean	95% Forecast interval	99% Forecast interval	Mean	95% Forecast interval	99% Forecast interval
GMST (°C)	2050	1.97	1.62–2.31	1.52–2.42	1.66	1.34–1.97	1.24–2.07
	2100	3.28	2.46–4.10	2.20–4.36	1.88	1.43–2.33	1.29–2.48
GMSL (mm)	2050	246.72	223.72- 269.72	216.49–276.95	229.67	207.96–251.38	201.13–258.21
	2100	655.25	556.68–753.82	525.70–784.80	531.23	471.52–590.95	452.76–609.71

	Year	SSP1-1.9	SSP1-2.6	SSP 2–4.5	SSP3-7.0	SSP4-6.0	SSP5-8.5
GMST (°C)	2050	1.43	1.66	2.02	2.32	2.17	2.43
	2100	1.04	1.44	2.81	5.09	3.54	6.79
GMSL (mm)	2050	216.15	229.47	250.33	268.39	259.03	271.93
	2100	453.92	507.36	638.03	805.73	701.11	921.64

### Forecast of regional mean sea level (RMSL)

**Figure 4:**
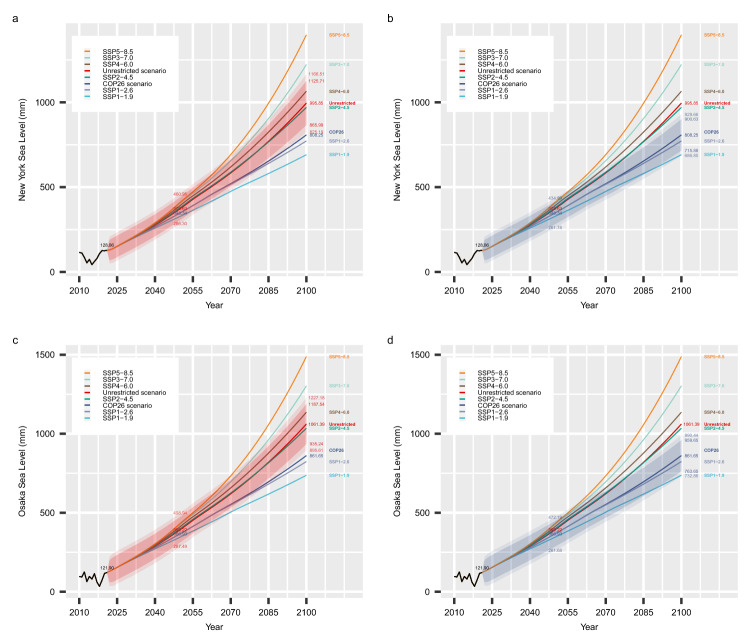
Regional mean sea level (RMSL) rise projections. The New York City (**a**,**b**) and Osaka (**c**,**d**) regional sea level rise projections under the unrestricted scenario (red), the COP26 scenario (grey), SSP5-8.5 (orange), SSP3-7.0 (light-green), SSP4-6.0 (brown), SSP2-4.5 (dark-green), SSP1-2.6 (light-grey) and SSP1-1.9 (light-blue) from now till 2100, the uncertainties (forecast interval) under the unrestricted and the COP26 scenarios were shown by the red and grey shaded area. For unrestricted and COP26 forecast, values of the expected means and the 95% and 99% forecast intervals are shown for 2050 and 2100 respectively. Historical data before 2022 are shown in black. The projections of the regional mean sea level are based on the ARDL models in Method section.

Regional sea level rise along the coastal lines is of great concern to the public and policymakers. For each major coastal region, we use the ARDL model, a versatile time series regression model, to predict the regional mean sea level (RMSL) based on its previous levels in time as well as the global mean sea level (GMSL) at the same and the prior time points. We studied eight coastal regions in total, with the New York City and Osaka results presented below. The other six regions are included in the Supplementary Materials. With stepwise variable selection, *RMSL*_*t*−1_ and *GMSL*_*t*_ are selected as significant predictors for all eight regional models. Based on the ARDL models, forecasts for New York City and Osaka are shown in Table 2 and Fig. 4.

**Table 2:** Predicted mean and forecast interval (95% and 99%) for the regional mean sea level (RMSL) rise at New York City and Osaka by 2050 and 2100 respectively under unrestricted or COP26 restrictions on greenhouse gas emissions; and the predicted means of the regional mean sea level (RMSL) rise at New York City and Osaka by 2050 and 2100 under SSP1-1.9, SSP1-2.6, SSP2-4.5, SSP3-7.0, SSP4-6.0 and SSP5-8.5 scenarios. The RMSL baselines for New York City and Osaka are the respective mean values of the 20-year RMSL for the two regions from 1986 to 2005. The units of RMSL are mm.

	Year	Unrestricted scenario			COP26 scenario		
		Mean (mm)	95% Forecast interval	99% Forecast interval	Mean (mm)	95% Upper forecast interval	99% Upper forecast interval
New York	2050	373.63	307.18–440.07	286.30–460.95	348.34	282.48–414.20	261.78–434.89
	2100	995.85	865.99–1125.71	825.19–1166.51	808.25	715.88–900.63	686.85–929.66
Osaka	2050	393.22	312.77–473.67	287.49–498.94	366.93	286.84–447.02	261.68–472.19
	2100	1061.39	935.24–1187.54	895.61–1227.18	861.65	763.65–959.65	732.86–990.44

	Year	SSP1-1.9 (mm)	SSP1-2.6 (mm)	SSP 2–4.5 (mm)	SSP3-7.0 (mm)	SSP4-6.0 (mm)	SSP5-8.5 (mm)
New York	2050	328.16	348.01	379.08	405.96	392.03	411.23
	2100	691.30	772.42	970.29	1223.20	1065.67	1398.03
Osaka	2050	345.81	366.56	398.99	427.07	412.51	432.45
	2100	737.11	823.84	1034.80	1303.12	1136.31	1488.58

The mean of 20 years of regional sea levels from 1986 to 2005 was taken as the baseline for the regional mean sea levels. By 2050, the New York City regional mean sea level (NYRMSL) will increase to 373.63 mm above the baseline, or equivalently 245.56 mm above its 2021 level under the unrestricted scenario, and these will reach 995.85 mm or 867.79 mm respectively by 2100. Under the COP26 scenario, by 2050, NYRMSL will increase to 348.34 mm above the baseline, or equivalently 220.28 mm above its 2021 level, and by 2100, these will be 808.25 mm or 680.20 mm, respectively. Under SSP 5-8.5, which is the most extreme scenario, by 2050, NYRMSL will increase to 411.23 mm above the baseline, or equivalently 283.16 mm above its 2021 level, and by 2100 these will be 1398.03 mm or 1269.97 mm, respectively (Fig. 4a,b).

Under the unrestricted scenario, the Osaka regional mean sea level (OSARMSL) will increase to 393.22 mm above the baseline, or 271.32 mm above its 2021 sea level by 2050, and these will reach 1061.39 mm or 939.49 mm by 2100. Under the COP26 scenario, OSARMSL will increase to 366.93 mm above the baseline, or 245.03 mm above its 2021 level by 2050, and these will reach 861.65 mm or 739.75 mm, respectively by 2100. Under SSP 5-8.5, which is the most extreme scenario, in 2050, OSARMSL will increase to 432.45 mm above the baseline, or equivalently 310.55 mm above its 2021 level, and by 2100 these will be 1488.58 mm or 1366.68 mm, respectively (Fig. 4c,d).

Considering the tidal effect, storm surge, and other climate factors, the maximum regional sea level can be much higher than the yearly mean sea level. The New York City (1920–2019) and Osaka (1961–2020) hourly mean sea level data were gathered to compute the yearly sea level fluctuation (the difference between the yearly maximum sea level and the yearly mean sea level). On the average, the yearly highest sea level is 1536.93 mm above the yearly mean sea level for New York City, while for Osaka it is 1138.95 mm. The Augmented Dickey–Fuller (ADF) tests confirm that the yearly fluctuations are stationary. On average, based on these assumptions and calculations, under the unrestricted scenario, the yearly highest sea level in 2100 could reach 2662.64 mm for New York City and 2326.49 mm for Osaka. Under the COP26 restricted emission scenario, these will be 2437.56 mm for New York City and 2098.60 mm for Osaka.

**Figure 5:**
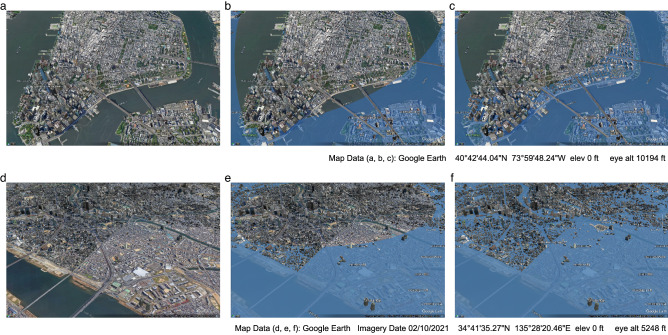
The 3D Google map simulation for regional mean sea level increase ranging from 0, to 1 and finally to 2 m for New York City (**a**–**c**) and Osaka (**d**–**f**) respectively, from its current level. According to our analysis, the New York City regional mean sea level will rise to 680.20 mm, 867.79 mm and 1269.97 mm higher than its current level under the COP26, unrestricted, and SSP5-8.5 scenarios respectively. The Osaka regional mean sea level will rise to 739.75 mm, 939.49 mm and 1366.68 mm higher than its current level under the COP26, unrestricted, and SSP5-8.5 scenarios, respectively.

For New York City, if the yearly mean sea level will increase to 997.65 mm above the 2021 level (the upper 95% forecast interval bound under the unrestricted scenario) by 2100, according to historical hourly data, on average there will be 45.54 days per year the daily highest sea level will be higher than 2 m above its current level as shown in Fig. 5c. The 45.54 days of severe flooding will reduce to 6.29 days if the COP26 restrictions on greenhouse gas emissions can be followed through. For Osaka, if the yearly mean sea level increases to 1065.64 mm above its 2021 level (the upper 95% forecast interval bound under unrestricted scenario) by 2100, according to historical hourly data, on average we will see 3.55 severe flooding days that the daily highest sea level will be higher than 2 m above its current level as shown in Fig. 5f, while this will reduce to 0.38 day under the COP26 restricted emission scenario. In summary, we found that the COP26 resolutions on anthropogenic greenhouse gas reduction would be helpful in controlling the global mean surface temperature to a certain extent, although such effects are not as pronounced in regulating global and regional sea level rises.

Figure 5 uses the program Google Earth Pro to present a 3D simulation for the chosen areas in 2100 under the different scenarios. Although the sea level rise conditions in the 3D maps do not exactly match individual greenhouse gas emission scenarios in our analysis due to limitations in the Google map resolution, these maps do sufficiently convey the range of consequences of sea level rise from the spectrum of greenhouse gas emission scenarios included in this study. Osaka will be more severely affected than New York City due to the difference in altitude between these two cities.

## Discussion

In this work, we presented a data driven approach to identify pathways leading to global warming and sea level rise. As expected, greenhouse gas emissions play a significant role in driving climate change. Furthermore, we found that the number of sunspots does not influence climate change significantly. We also found global surface temperature and humidity in a positive feedback loop with each other, while higher temperature induces higher sea level through the melting of sea ice and glaciers as well as ocean water thermal expansion. The melting glacier also has a direct hand in the melting of sea ice in addition to the higher temperature. Forecasting based on the pathway system reveals that without any intervention, global surface temperature will rise to a mean level of 3.28 °C above its pre-industrial level by 2100; whereas if the COP26 resolution on the reduction of anthropogenic greenhouse gas emission can be followed through, such increase would reduce to 1.88 °C, just short of the intended goal of 1.5 °C. The global sea level, however, will not be as drastically reduced by the COP26 resolution, with the global sea-level expected to rise an average of 448.81 mm above its 2021 mean following COP26 emission regulations, while 572.83 mm with no regulations. Regional sea level projections based on our models show that the increased global sea level will induce more frequent and severe flooding for the world’s coastal regions including heavily populated metropolis such as New York City and Osaka. This calls for added protective measures in infrastructure to ensure that urban areas are more resilient to extreme flooding and tidal surges. Lastly, the data driven findings for the unrestricted scenario are found to be highly consistent with the 2021 IPCC projections on climate change, underscoring the necessity to take drastic actions to mitigate future damages. The passage of the Inflation Reduction Act with $369bn (£305bn) budgeted for climate action through the US Congress, the largest investment in US history—has brought a glimmer of hope in this critical moment of our collective fight to ensure a sustainable earth and future.

Finally, we acknowledge that the main obstacle to our analysis is the limited length of historical data available. Our model is a linear system due to data limitation, while the real-world climate processes are often non-linear. However, the short-term prediction accuracy of our system has been validated by back-testing based on historical data. Moving forward, we will update our forecasting system as more data are available. Furthermore, we will include more climate features such as ocean processes and paleoclimate time series as these data are increasingly procured and validated as technology progresses^68^. We are hopeful to have a more panorama view of the climate change on earth through this incremental process.

## Methods

### Data

In this study, the following climate-related variables with yearly frequency from 1950 to 2021 were collected from multiple sources:

1. Global Mean Sea Level (GMSL) in mm was gathered from the mean of two data sources^69,70^. The data for the year 2021 were collected and calibrated from GSFC (2021)^2^.
2. Glaciers and Ice Sheets Mass Balance (Mass) in Gt (Gigaton) was estimated by two methods: (i) the product of global mean glacier mass in water equivalence from the world glacier monitoring service [1950–2021]^71^ and the total glaciers and ice sheets area^72^; (ii) Summation of 17 mountain glaciers mass balance^73^, Greenland and Antarctica ice sheet mass balance [2002–2020]^74^, with missing values imputed by Kalman filter^75^; overlapping period of estimation (i) and (ii) were calibrated by the average values.
3. Arctic Sea Ice August Extent (SeaIce) in Mkm2 was gathered from two sources: (i) observed August Arctic sea-ice extent [1870–2008]^76^, and (ii) observed north hemisphere sea ice extent in August [1979–2021]^77^. The least squares method was adopted to calibrate and combine these two data sources.
4. Global Surface Temperature (with sea ice area measured by the air above sea ice) (GMST) in °C was obtained from Rohde (2020)^1^.
5. Global Specific humidity (Humidity) in kg/kg (mass of water vapor per kilogram of moist air)^78^.
6. Greenhouses gases: CO_2_ [1950–1957]^79^ [1958–2021]^80^, CH_4_ [1950–1983]^81^ [1984–2021]^82^, and N_2_O [1950–1977]^81^ [1978–2021]^83^. Global warming potential (GWP), which is measured by units of CO_2_ equivalents in the environmental impacts, is adopted to analyze the effect of greenhouse gases in this study. The formulation to calculate GWP is shown in Eq. (1), following Intergovernmental Panel on Climate Change (IPCC) reports^84^.
  1 $$GWP=C{O}_{2}+28C{H}_{4}+265{N}_{2}O$$
7. Greenhouse gases concentration projection for CO_2_, CH_4_ and N_2_O [2022–2100] under SSP scenarios^66,67^.
8. Sunspot Number (SSN)^85^.
9. Regional Mean Sea Level (RMSL) data were obtained from the Permanent Service for Mean Sea Level^86,87^: New York City RMSL is obtained from the Battery station, and Osaka RMSL is obtained from the Osaka station.
10. New York City Hourly Water Level data were collected from the University of Hawaii Sea Level Center^70^, and Osaka Hourly Water Level data were collected from Japan Oceanographic Data Center^88^.

Augmented Dickey–Fuller (ADF) test results confirm all the variables are integrated of order 1, i.e. I(1) processes, based on historical data analysis.

### Path analysis model identification and estimation for global mean surface temperature (GMST) and global mean sea level (GMSL)

Structural equation modeling (SEM) refers to a family of multivariate procedures designed to infer causal relationships among a set of variables^89^. It can be viewed as a system of multivariate regression equations with variables that can serve as both independent and dependent variables. Latent variables are also common although in this work we did not use any latent variables. Inference of SEM usually focuses on estimating the model implied population covariance matrix through the likelihood function:

2 $${F}_{ML}=log|\Sigma (\theta )|+tr(S{\Sigma (\theta )}^{-1})-{\mathrm{log}}|S|-q,$$

where $\Sigma (\theta )$ is the model implied covariance matrix with $\theta$ representing model parameters including path coefficients, *S* is the sample variance–covariance matrix, and *q* is the number of parameters to be estimated. The unified structural equation model (uSEM)^4^, also known as Vector Autoregression-Structural Equation Modeling (VAR-SEM) or the Dynamic SEM modeling can be used to analyze the proposed causal associations among a set of time-series variables by incorporating both the contemporaneous relations and the longitudinal relations, simultaneously. Contemporaneous relations reflect relationships between variables at the same time point, while longitudinal temporal relations are defined as relationships between variables at different time points.

Variable selection for the hypothesized full uSEM model in this work was done in two approaches:

1. A system-wise variable selection based on the entire set of uSEM equations via the regularized unified structural equation modeling (RuSEM) method^51^, where regularization is done through the adaptive Lasso^90^.
2. An equation-wise variable selection based on each individual uSEM equation, which can also be viewed as an autoregressive distributed lags (ARDL) model. The variable selection for each ARDL was done through the backward stepwise variable selection method.

The adaptive LASSO^90^ penalizes parameters after each parameter is scaled by the un-penalized maximum likelihood estimators (MLE) in the following equation:

3 $${F}_{\text{alasso }}={F}_{ML}+\lambda \|{{\theta }_{ML}^{-1}*{\theta }_{pen}}\|_{1},$$

where *λ* is the regularization parameter with a common initialization value of 0.1.

For equation-wise variable selection, each individual ARDL model has the following general form:

4 $${y}_{t}={\beta }_{0}+{\beta }_{1}{y}_{t-1}+\cdots +{\beta }_{p}{y}_{t-p}+{\delta }_{0}{x}_{t}+{\delta }_{1}{x}_{t-1}+\cdots +{\delta }_{q}{x}_{t-q}+{\epsilon }_{t},$$

where *y*_*t*_ is the endogenous variable at time t, *x*t is the exogenous variable at time t, ${\beta }_{0}$ is the intercept, ${\beta }_{i},\; i\in \{1,\dots ,p\}$ and ${\delta }_{j},\; j\in \{0,\ldots ,q\}$ are the coefficients. The Akaike information criterion (AIC) is used to de-select unnecessary paths in the backward stepwise variable selection.

For our work, the final path model selected based on the system-wise and the equation-wise variable selection methods happen to be identical—signifying the robustness of our climate pathway result. The final path model, namely, the final refitted uSEM using only the significant pathways identified, is represented in the matrix form as follows, which can also be viewed as a system of ARDL time series regression equations:

5 $$\begin{aligned} \left[\begin{array}{l}Humidit{y}_{t}\\ TEM{P}_{t}\\ Mas{s}_{t}\\ Sea{Ice}_{t}\\ GMS{L}_{t}\end{array}\right]&= \left[\begin{array}{ccccc}0& 0& 0& 0& 0\\ 0.443& 0& 0& 0& 0\\ 0& -0.022& 0& 0& 0\\ 0& -0.335& 0& 0& 0\\ 0& 0.089& 0& 0& 0\end{array}\right]\left[\begin{array}{l}{Humidity}_{t}\\ TEM{P}_{t}\\ Mas{s}_{t}\\ {SeaIce}_{t}\\ GMS{L}_{t}\end{array}\right]\\ &\quad + \left[\begin{array}{ccccc}0.433& 0.374& 0& 0& 0\\ -0.329& 0.683& 0& 0& 0\\ 0& 0& 1.008& 0& 0\\ 0& 0& 0.320& 0.347& 0\\ 0& 0& -0.228& -0.097& 0.606\end{array}\right]\left[\begin{array}{l}{Humidity}_{t-1}\\ TEM{P}_{t-1}\\ Mas{s}_{t-1}\\ {SeaIce}_{t-1}\\ GMS{L}_{t-1}\end{array}\right]\\ &\quad + \left[\begin{array}{l}0\\ 0.226\\ 0\\ 0\\ 0\end{array}\right]\left[GW{P}_{t-1}\right]+\left[\begin{array}{l}0.034\\ 0.029\\ -0.052\\ -0.026\\ 0.047\end{array}\right] \end{aligned}$$

### GWP forecasts under COP26

The 26th United Nations Climate Change Conference (COP26), held in Glasgow, UK, from 31 October to 13 November 2021, has reached two key resolutions to reduce the anthropogenic greenhouse gas emissions: (1) to reduce the anthropogenic CO_2_ (carbon dioxide) emission by 45% by 2030, compared to its 2010 levels, and (2) to reduce the anthropogenic CH_4_ (methane) emissions by 30% by 2030, compared with its 2020 levels.

It is assumed that without anthropogenic emissions, the greenhouse concentration should remain stable, the same as last year. The global CO_2_ concentration level in 2010 and 2009 was measured at 389.89 parts per million (ppm) and 387.34 ppm respectively, featuring a yearly increase of 2.54 ppm. With the assumption, we conclude all 2.54 ppm increase is anthropogenic, that is, due to human activities. Per COP26, for 2030, the expected concentration increase caused by anthropogenic emission of CO_2_ will be $(1 - 0.45) * 2.54 \approx 1.40$ ppm. To simplify the matter, we have adopted a linear decrease, which means every year we will see the amount of emission decrease stays constant (we can also use other models such as exponential, which will not significantly change our results). The increase in CO_2_ concentration in 2021 is 2.12 ppm. Therefore, if we can follow through the COP26 resolution, the annual increase of anthropogenetic CO_2_ concentration, from 2022 to 2030, in a linear decreasing pattern, will be (2.04, 1.96, 1.88, 1.80, 1.72, 1.64, 1.56, 1.48, 1.40) ppm. After 2030, it is assumed that the increase of CO_2_ concentration will continue decreasing at the same rate until dropping to zero increase.

The global CH_4_ concentration levels in 2020 and 2019 were measured at 1879.11 parts per billion (ppb) and 1866.60 ppb respectively, featuring a yearly increase of 12.51 ppb. With the assumption above, we conclude all 12.51 ppb is anthropogenic, that is, due to human activities. Per COP26, for 2030, the expected concentration increase caused by anthropogenic emission of CH_4_ will be $(1 - 0.3) \times 12.51 \approx 8.76$ ppb. To simplify the matter, we have adopted a linear decrease, which means every year we will see the amount of emission decrease stays constant (we can also use other models such as exponential, which will not significantly change our results). The increase in CH_4_ concentration in 2021 is 16.52 ppb. Therefore, if we can follow through the COP26 resolution, the annual increase of anthropogenetic CH_4_ concentration, from 2022 to 2030, in a linear decreasing pattern, will be (15.66, 14.79, 13.93, 13.07, 12.21, 11.34, 10.48, 9.62, 8.76) ppb. After 2030, it is assumed that the increase of CH_4_ concentration will continue decreasing at the same rate until dropping to zero increase.

Since no constraints were proposed for N_2_O, it is assumed the N_2_O concentration will increase following its historical trend, which is well fitted by an ARIMA(1,1,1) model. The uncertainty of the forecasted GWP level associated with the COP26 scenario was calculated under the assumption that the coefficient of variation ($CV = \sigma /\mu$, where $\sigma$ is the standard deviation and $\mu$ is the mean) remains constant. The standard deviation in the COP26 scenario is the corresponding standard deviation under the unrestricted scenario times the ratio of the mean of these two scenarios ($\sigma_{C} = \sigma_{N} \mu_{C} /\mu_{N}$). The final identified uSEM, the time series forecasts of GWP and its uncertainties, are used to forecast the future trend and uncertainties of GMST and GMSL until 2100 under the unrestricted scenario, the COP26 restriction scenario and the SSP scenarios. Meanwhile, backtesting tests using historical data were conducted to validate the forecast models. The backtesting used a 9:1 ratio of the training set to the test set. For the test data, both one-step ahead (predictors updated with true observed values at each time step) and multi-step ahead forecasting (predictors updated with forecasted values at each time step) were performed. Both results show that all the true values fall within the 99% forecast intervals validating the robustness of our model. Details are available in the Supplementary Materials.

### Regional mean sea level (RMSL) forecasts

To forecast the RMSL, we have adopted the ARDL model with the stepwise variable selection method. The ARDL variable selection results are provided in Eq. (6) below:

6 $$\begin{aligned} NYRMSL_{t} & = 0.2477 + 0.2640NYRMSL_{t - 1} + 1.1248GMSL_{t} \\ OSARMSL_{t} & = - 2.4039 + 0.4461OSARMSL_{t - 1} + 0.9129GMSL_{t} , \\ \end{aligned}$$

where $NYRMSL_{t}$ is the regional mean sea level for New York City in year *t*, $OSARMSL_{t}$ is the regional mean sea level in Osaka in year *t*, $GMSL_{t}$ is the global mean sea level in year *t*. Results for the other six coastal locations examined are available in the Supplementary Materials. To reduce the impact of missing values in regional sea level data, we used a longer time span (1950–2015) for the training data when backtesting regional models. Model validation is confirmed by the backtesting results, showing that all the true values fall within the predicted range for all regions.

### Consistency with physics-based models and forecasts

Although the data driven uSEM pathway modeling does not rely on physical theories and models, it still comes as a relief when we found our results highly consistent with the recent forecasts based largely on scientific theories. The IPCC 2021 climate change report^91^ has shown projections for global mean surface temperature (GMST) and global mean sea level (GMSL) rise under different scenarios. As the IPCC reports use a different method to assess greenhouse gas emissions than our model, we use Meinshausen et al.^92^ to compare the scenarios of our model with those of the IPCC. According to Meinshausen et al.^92^, the GWP concentration in 2100 will be 568.96 ppm in the SSP 1-2.6 scenario and 749.91 ppm in the SSP 2-4.5 scenario, which is comparable to our COP26 scenario forecast of 631.60 ppm and the unrestricted scenario forecast of 821.02 ppm, respectively. According to IPCC 2021, GMST under SSP2-4.5 scenario will be approximately 2.7 (2.1–3.5) °C by 2100, which is comparable to our unrestricted scenario projection of 3.28 (2.46–4.10) °C, and 1.8 (1.3–2.4) °C under SSP 1-2.6 scenario, which is close to our COP26 projection of 1.88 (1.43–2.33) °C under COP26 scenario. The GMSL is anticipated to rise by 464–784 mm under SSP2-4.5 scenario and 344–644 mm under SSP1-2.6 scenario in 2100 according to IPCC 2021, which agree well with our projection of 655.25 (556.68–753.82) mm for the unrestricted scenario and 531.23 (471.52–590.95) mm for the COP26 scenario. All estimates are calibrated to our baselines as mentioned before.

Per physics driven forecasts conducted by our group^8^, the GMSL will be 2.3 feet (701.04 mm) by 2100, which is highly consistent with our data driven forecast (655.25 mm) for the unrestricted scenario. The 95% confidence interval of the New York City regional sea level is estimated to range from 1.6 feet (487.68 mm) to 4.1 feet (1249.68 mm) above the baseline based on the physics driven models^8^, while the 95% confidence interval based on our data driven models is 865.99–1125.71 mm, residing completely inside the former confidence interval.

From a climate science point of view, the relationship between temperature and the specific humidity is not linear and outlined by the August–Roche–Magnus (ARM) formula^93,94^ as follows:

7 $$\begin{aligned} e_{s} & = 6.1094{\text{exp}}\left( {\frac{17.625T}{{T + 243.04}}} \right) \\ q & = \beta *RH*e_{s} /\left( {p - \left( {1 - \beta } \right)e_{s} } \right), \\ \end{aligned}$$

where $e_{s}$ is the saturation vapor pressure in hPa, *T* is the temperature in Celsius degree (*T* = GMST + 14.105 °C), *q* is the specific humidity in kg/kg, RH is the relative humidity ranging from 0 to 1, *p* is the atmospheric pressure in hPa (≈ 1013.25), and $\beta$ is a constant coefficient (≈ 0.622). Although the ARM formula indicates a nonlinear relationship between temperature (*T*) and humidity (*q*), we can prove that this nonlinear relation can be well approximated by a linear relationship between *T* and *q* via Taylor Theorem, thus validating the sufficiency of our linear model structure.

According to the Taylor expansion, a function *q*(*T*) that is infinitely differentiable at $T = T_{0}$ is equal to a power series of the form $q\left( T \right) = \sum\nolimits_{i = 0}^{\infty } {a_{i} \left( {T - T_{0} } \right)^{i} }$, where $a_{i} = \frac{{q^{\left( n \right)} \left( {T_{0} } \right)}}{i!}$. Therefore, we can write the ARM as:

8 $$q(T) = a_{0} + a_{1} (T - T_{0} ) + R_{2} (T).$$

According to the Taylor Theorem, the error term $R_{2} (T)$ satisfies $R_{2} (T) = \frac{{q^{(2)} (T^{\prime})}}{2!}(T - T_{0} )^{2}$ for some $T^{\prime}$ in the range of *T* and *T*_0_. Choosing $T_{0} = 14.62$ and using the first two terms as the linear representation $q_{lin} (T)$ of the ARM, we can show that the difference between the ARM formula ($q_{ARM} (T)$) and the linear representation ($q_{lin} (T)$) is:

9 $$\begin{array}{*{20}r} \hfill {\left| {q_{ARM} (T) - q_{lin} (T)} \right| = \left| {R_{2} (T)} \right| < 1.94 \times 10^{ - 4} RH < 1.94 \times 10^{ - 4} , \quad {\text{for }}T \in \left[ {13.80,\;17.50} \right].} \\ \end{array}$$

The temperature range includes the observed data from 1950 and the 99% prediction intervals of our model. Therefore, a linear representation is sufficient in approximating the non-linear relationship between GMST and Humidity given by the ARM formula. A numeric comparison is provided in the Supplementary Materials.

### Reduced form and structural form of the uSEM

There are two equivalent ways to present the unified structural equation model (uSEM), the reduced form and the structural form. The main differences are: (1) In the reduced form, the right-hand (regressor) side features only terms at previous lags such t − 1, etc.; while the structural form includes both terms at time *t*, and at the previous lags, *t* − 1, etc., on the right-hand side. (2) For the reduced form, the errors of the uSEM are correlated, while for the structural form, the errors are uncorrelated.

In our work, we have shown the structural form for the pathway model, which is more intuitive in understanding the temporal autocorrelations as follows:

$$\begin{gathered} \left\{ {\begin{array}{*{20}l} {Humidity_{t} = 0.034 + 0.433 Humidity_{t - 1} + 0.374 GMST_{t - 1} + \epsilon_{{1{\text{t}}}} } \\ {GMST_{t} = 0.029 - 0.329 Humidity_{t - 1} + 0.683 GMST_{t - 1} + 0.443 Humidity_{t} + 0.226 GWP_{t - 1} + \epsilon_{{2{\text{t}}}} } \\ \end{array} } \right. \hfill \\ \hfill \\ \end{gathered}$$

where $corr(\epsilon_{{1{\text{t}}}} ,\;\epsilon_{{2{\text{t}}}} ) \approx 0$. We have applied the structural form for prediction as it is easier to compute as well thanks to the uncorrelated error structure. Worth to mention, the negative coefficient of from $Humidity_{t - 1}$ to $GMST_{t}$ is caused by the multi-collinearity between $Humidity_{t - 1}$ and $Humidity_{t}$, the interactive effect between Humidity and GMST are positive overall with both $Humidity_{t - 1}$ and $Humidity_{t}$.

Additionally, $GMST_{t}$ and $Humidity_{t}$ affect each other simultaneously actually, which is represented by a correlation between the error terms {$\epsilon_{1t} ,\; \epsilon_{2t}^{{\prime}}$} in the equivalent reduced form shown below:

$$\left\{ {\begin{array}{*{20}l} {Humidity_{t} = 0.034 + 0.433 Humidity_{t - 1} + 0.374 GMST_{t - 1} + \epsilon_{1t} } \\ {GMST_{t} = 0.044 - 0.138 Humidity_{t - 1} + 0.849 GMST_{t - 1} + 0.226 GWP_{t - 1} + \epsilon_{{2{\text{t}}}}^{{\prime}} } \\ \end{array} } \right.$$

where the estimated correlation, $corr(\epsilon_{{1{\text{t}}}} ,\;\epsilon_{{2{\text{t}}}}^{{\prime}} ) \approx 0.869$.

## Supplementary Information

Supplementary Information.

## Data Availability

Datasets containing Global Mean Sea Level matched for this study are available at Climate.gov: https://www.climate.gov/sites/default/files/Climate_dot_gov_dashboard_SeaLevel_Jan2021update.txt and https://climate.nasa.gov/vital-signs/sea-level/ (Retrieved on May 20th, 2022). Datasets containing Glaciers and Ice Sheets Mass Balance for this study are available at World Glaciers Monitoring Service: https://wgms.ch/global-glacier-state/, Datasets underlying the publication: Global Glacier Mass Loss During the GRACE Satellite Mission: 10.4121/13663433.v1, and NASA’s GRACE and GRACE Follow-On satellites: 10.5067/TEMSC-3MJ62 (Retrieved on May 20th, 2022). Datasets containing Arctic Sea Ice August Extent for this study are available at National Snow and Ice Data Center: https://nsidc.org/arcticseaicenews/sea-ice-tools/ and http://poles.tpdc.ac.cn/en/data/fb3cfed6-9d2f-4664-b30b-c1c41d253ddb/ (Retrieved on May 20th, 2022). Datasets containing Global Mean Surface Temperature for this study are available at Berkeley Earth: http://berkeleyearth.org/data/ (Retrieved on May 20th, 2022). Datasets containing Global Specific Humidity for this study are available at Copernious: https://cds.climate.copernicus.eu/#!/home (Retrieved on Jan 29th, 2022). Datasets containing CO_2_ for this study are available at CDIAC https://cdiac.ess-dive.lbl.gov/trends/co2/lawdome.html and Scripps CO_2_ Program https://scrippsco2.ucsd.edu/data/atmospheric_co2/mlo.html (Retrieved on May 20th, 2022). Datasets containing CH_4_ for this study are available at NOAA: https://www.ncei.noaa.gov/access/paleo-search/study/9959 and https://gml.noaa.gov/ccgg/trends_ch4/ (Retrieved on May 20th, 2022). Datasets containing N_2_O for this study are available at NOAA: https://www.ncei.noaa.gov/access/paleo-search/study/9959 and https://gml.noaa.gov/hats/combined/N2O.html (Retrieved on May 20th, 2022). Datasets containing furture concentrations for CO_2_, CH_4_ and N_2_O under SSP scenarios at University of Melbourne: https://greenhousegases.science.unimelb.edu.au/#!/ghg?mode=downloads (Retrieved on Nov. 27th, 2022). Datasets containing Sunspot Number for this study are available at SILSO: https://www.sidc.be/silso/datafiles (Retrieved on May 20th, 2022). Datasets for all the Regional Mean Sea Level (RMSL) data is obtained from the Permanent Service for Mean Sea Level (PSMSL): http://www.psmsl.org/data/obtaining/ (Retrieved on May 27th, 2022). Datasets containing New York City hourly water levels are available at https://uhslc.soest.hawaii.edu/datainfo/ and Osaka at https://jdoss1.jodc.go.jp/vpage/tide.html (Retrieved on Apr 29th, 2022).

## Code availability

The R codes used for the analysis and graphics of this paper are available upon request.
