# Supplementary material for: Data driven pathway analysis and forecast of global warming and sea level rise
Source: Sci Rep. 2023 Apr 4;13:5536. doi: 10.1038/s41598-023-30789-4 (PMC10073234; doi:10.1038/s41598-023-30789-4)
Supplement: Supplementary file 1 — Supplementary Information. [file 41598_2023_30789_MOESM1_ESM.docx]

Data Driven Pathway Analysis and Forecast of Global Warming and Sea Level Rise

Supplementary Information

Figure S1: Overview of climate variables included in this study. Line plots of 9 climate factors from 1950-2020 in yearly frequency are provided as follows: a. Global Mean Sea Level (GMSL) in millimeters (mm); b. Glaciers and Ice Sheets Mass (Mass) in gigatons (Gt); c. Arctic Sea Ice Extent in August (SI) in million kilometers square (Mkm2); d. Global Mean Surface Temperature (GMST) in Celsius degree (°C); e. Global Specific humidity (Hm) in kilogram per kilogram (kg/kg); f. CO_2_ atmosphere concentration (CO_2_) in parts per million (ppm); g. CH4 atmosphere concentration (CH_4_) in parts per billion (ppb); h. N_2_O atmosphere concentration (N_2_O) in parts per billion (ppb); i. Sunspot Number (SSN).

Table S1: Correlation analysis demonstrates that the concentrations of the three greenhouse gases in the atmosphere are highly correlated, which could contribute to multicollinearity. Hence, the global warming potential (GWP) is adopted to represent the global greenhouse gas level instead of the individual gases.

| **Correlation** | **CO_2_** | **CH_4_** | **N_2_O** |
| --- | --- | --- | --- |
| CO_2_ | 1.000 | 0.931 | 0.998 |
| CH_4_ | 0.931 | 1.000 | 0.944 |
| N_2_O | 0.998 | 0.944 | 1.000 |

Table S2: The augmented Dickey–Fuller test (ADF) tests were performed to determine the order of climatic factor time series. All the climate factors are I(1) time series, as all the original data are not stationary at the significance level 0.05 (p-values > 0.05) while all the once difference data are stationary at significance level 0.05 (p-values 0.05).

| **Variables** | **ADF test p-value** |
| --- | --- |
| GMSL | 0.87 |
| ΔGMSL | 0.01 |
| Mass | 0.99 |
| ΔMass | 0.01 |
| SeaIce | 0.21 |
| ΔSeaIce | 0.01 |
| TEMP | 0.08 |
| ΔTEMP | 0.01 |
| Humidity | 0.06 |
| ΔHumidity | 0.01 |
| GWP | 0.93 |
| ΔGWP | 0.01 |

Table S3: ADF tests for the ARDL model residuals with p-values less than 0.05 showing all the model residuals are stationary.

| **Residuals** | **ADF test p-value** |
| --- | --- |
| GMSL | 0.01 |
| Mass | 0.01 |
| SeaIce | 0.02 |
| TEMP | 0.01 |
| Humidity | 0.01 |

Table S4: Shapiro–Wilk (normality) tests for model residuals. The p-values indicate that all model residuals are normal except for SeaIce.

| **Residuals** | **Shapiro–Wilk test p-value** |
| --- | --- |
| GMSL | 0.52 |
| Mass | 0.33 |
| SeaIce | 0.00 |
| TEMP | 0.91 |
| Humidity | 0.88 |

Figure S2: Global warming potential (GWP) components. The green area represents the contribution of carbon dioxide (CO_2_); the blue area represents nitrogen oxide (N_2_O), and the red area represents methane (CH_4_). The y-axis represents the cumulative concentration of greenhouse gases in the atmosphere.

Figure S3: Calibration of Global Mean Sea Level (GMSL), Arctic August Sea Ice Extent (SeaIce) and Glaciers and Ice Sheets Mass Balance data (Mass). a. Calibration results of GMSL: the blue line represents data from source 1; the red line represents the data from source 2; the green line represents the calibrated data from source 3; the black line represents the calibration results. b. Calibration results of Sea Ice: the blue line represents data from source 1; the red line represents data from source 2; the black line represents the calibration results. c. Two methods for estimating Mass (the blue line and the red line), with the black line representing the calibration results.

Figure S4: Comparison of the humidity models. The black line is the true observation; the red line is the estimation of ARM formula in physics-based model (relative humidity (RH) is estimated by the least squares method), while the blue line is the estimation of the uSEM model.

Figure S5: Backtesting results based on the uSEM model. Both the one-step ahead forecasts and the multi-step ahead forecasts were conducted. The grey line represents the predicted values, while the black line represents the actual values. The dark grey shaded area represents the 95% prediction interval, while the light grey shaded area represents the 99% prediction interval. The backtesting results indicate that all actual GMST and GMSL values fall within the 99% prediction interval. It demonstrates the models’ validity.

Figure S6: Backtesting results for New York and Osaka based on the ARDL models. Backtesting results based on the proposed model, with parameters fitted by the training data. One-step ahead forecast (predictor will be updated with true value observed) and multi-step ahead forecast (predictor will not be updated and use the forecast value) are conducted. The Grey line is the forecast values; the black line is the true values; the dark grey shaded area is the 95% prediction interval while the light grey shaded area is the 99% prediction interval. The backtesting results show that all the true values fall within the 99% forecast interval for New York and Osaka indicating the models are valid.

Figure S7: Comparative analysis of Osaka and four stations in Japan. The red line represents the annual sea level at Osaka, while the blue line represents the average annual sea level at four stations in Japan. The average sea level information is provided by Japan Meteorological Agency^1^. The graph indicates that Osaka’s annual sea level data from 1965 to 1969 is anomalous. Thus, this paper only used Osaka data after 1970.

Table S5: Data sources of the regional sea level data

| **Locations** | **Stations** |
| --- | --- |
| New York (NY) | New York (the Battery), New York |
| Osaka (OSA) | Osaka, Japan |
| Shanghai (SH) | Kanmen, WenZhou, China; Lusi, Nantong, China |
| Hawaii (HI) | Hilo, Hawaii |
| Ionian Sea Coast(ION) | Marsaxlokk, Malta; Catania, Italy; Capo Passero, Italy; Levkas, Greece; Katakolon, Greece |
| London (LON) | Newlyn, UK; Southend, UK |
| Ijmuiden (IJ) | Ijmuiden, Netherland |
| Dieppe (DP) | Dieppe, France |
| The data are downloaded from the Permanent Service for Mean Sea Level^2,3^. New York, Osaka and Hawaii have the data for 2021, while others lack data for 2021. The mean value is used when data is from multiple stations. | |

After variable selections, the models for the six regional sea levels are as follows:

$$\begin{matrix} SHRMSL_{t} & =3.5884+0.2583SHRMSL_{t-1}+1.1493GMSL_{t} \\ HIRMSL_{t} & =-3.2083+0.3412HIRMSL_{t-1}+0.9582GMSL_{t} \\ IONRMSL_{t} & =2.7948+0.5442IONRMSL_{t-1}+0.5348GMSL_{t} \\ LONRMSL_{t} & =1.6768+0.3686LONRMSL_{t-1}+0.5483GMSL_{t} \\ IJMRMSL_{t} & =-5.8125+0.3531IJMRMSL_{t-1}+0.5994GMSL_{t} \\ DPRMSL_{t} & =-3.6966+0.3714DPRMSL_{t-1}+1.1565GMSL_{t}, (1) \end{matrix}$$


Figure S8: Backtesting results for six regional sea levels based on the ARDL models. The configuration is the same for Figure S6. The backtesting results show that all actual values fall within the 99% prediction interval demonstrating the validity of the models.

Table S6: Regional mean sea level forecast results for New York, Osaka and six other regions based on the ARDL models under unrestricted scenario and COP26 scenario.

|  | | **Unrestricted Scenario** | | | **COP 26 Scenario** | | |
| --- | --- | --- | --- | --- | --- | --- | --- |
| **Region** | **Year** | **Mean (mm)** | **95% Forecast Interval** | **99% Forecast Interval** | **Mean (mm)** | **95% Forecast Interval** | **99% Forecast Interval** |
| New York | 2050 | 373.63 | 307.18-440.07 | 286.30-460.95 | 348.34 | 282.48-414.20 | 261.78-434.89 |
|  | 2100 | 995.85 | 865.99-1125.71 | 825.19-1166.51 | 808.25 | 715.88-900.63 | 686.85-929.66 |
| Osaka | 2050 | 393.22 | 312.77-473.67 | 287.49-498.94 | 366.93 | 286.84-447.02 | 261.68-472.19 |
|  | 2100 | 1061.39 | 935.24-1187.54 | 895.61-1227.18 | 861.65 | 763.65-959.65 | 732.86-990.44 |
| Shanghai | 2050 | 383.44 | 332.63-434.25 | 316.67-450.22 | 357.78 | 307.77-407.79 | 292.05-423.50 |
|  | 2100 | 1014.40 | 889.75-1139.05 | 850.58-1178.22 | 824.13 | 741.26-907.01 | 715.22-933.05 |
| Hawaii | 2050 | 348.85 | 267.73-429.97 | 242.24-455.46 | 325.09 | 244.34-405.85 | 218.96-431.23 |
|  | 2100 | 940.19 | 813.44-1066.94 | 773.61-1106.77 | 762.45 | 663.85-861.04 | 632.87-892.03 |
| Ionian | 2050 | 286.12 | 214.33-357.91 | 191.77-380.47 | 268.00 | 196.35-339.64 | 173.84-362.15 |
|  | 2100 | 760.10 | 666.23-853.97 | 636.74-883.46 | 619.45 | 539.64-699.27 | 514.56-724.35 |
| London | 2050 | 213.46 | 164.96-261.95 | 149.72-277.19 | 199.35 | 151.06-247.64 | 135.88-262.82 |
|  | 2100 | 566.30 | 491.98-640.62 | 468.63-663.98 | 460.38 | 402.05-518.71 | 383.72-537.04 |
| Ijmuiden | 2050 | 216.17 | 147.51-284.83 | 125.94-306.40 | 201.07 | 132.58-269.56 | 111.06-291.08 |
|  | 2100 | 592.77 | 500.80-684.74 | 471.90-713.64 | 479.64 | 402.47-556.80 | 378.23-581.04 |
| Dieppe | 2050 | 440.58 | 353.21-527.95 | 325.75-555.40 | 410.72 | 323.85-497.58 | 296.55-524.88 |
|  | 2100 | 1187.97 | 1040.41-1335.53 | 994.04-1381.90 | 963.63 | 852.64-1074.62 | 817.77-1109.49 |
| The RMSL baselines are the average RMSL values for the eight regions over a 20-year period from 1986 to 2005. | | | | | | | |

Table S7: Regional mean sea level forecast results for New York, Osaka and six other regions based on the ARDL models under SSP scenarios.

| **Region** | **Year** | **SSP1-1.9 (mm)** | **SSP1-2.6 (mm)** | **SSP 2-4.5 (mm)** | **SSP3-7.0 (mm)** | **SSP4-6.0 (mm)** | **SSP5-8.5 (mm)** |
| --- | --- | --- | --- | --- | --- | --- | --- |
| New York | 2050 | 328.16 | 348.01 | 379.08 | 405.96 | 392.03 | 411.23 |
|  | 2100 | 691.30 | 772.42 | 970.29 | 1223.20 | 1065.67 | 1398.03 |
| Osaka | 2050 | 345.81 | 366.56 | 398.99 | 427.07 | 412.51 | 432.45 |
|  | 2100 | 737.11 | 823.84 | 1034.80 | 1303.12 | 1136.31 | 1488.58 |
| Shanghai | 2050 | 337.31 | 357.45 | 388.97 | 416.25 | 402.11 | 421.60 |
|  | 2100 | 705.52 | 787.78 | 988.46 | 1245.00 | 1085.20 | 1422.33 |
| Hawaii | 2050 | 306.09 | 324.78 | 354.01 | 379.30 | 366.19 | 384.23 |
|  | 2100 | 651.63 | 728.60 | 916.17 | 1155.49 | 1006.53 | 1320.92 |
| Ionian | 2050 | 253.34 | 267.71 | 290.17 | 309.62 | 299.52 | 313.25 |
|  | 2100 | 531.75 | 593.05 | 741.75 | 930.11 | 813.19 | 1060.27 |
| London | 2050 | 188.06 | 199.16 | 216.53 | 231.56 | 223.77 | 234.48 |
|  | 2100 | 394.34 | 440.24 | 552.04 | 694.58 | 605.88 | 793.11 |
| Ijmuiden | 2050 | 188.99 | 200.87 | 219.45 | 235.53 | 227.19 | 238.66 |
|  | 2100 | 409.10 | 458.11 | 577.51 | 729.81 | 635.02 | 835.08 |
| Dieppe | 2050 | 386.80 | 410.31 | 447.08 | 478.91 | 462.41 | 485.08 |
|  | 2100 | 823.77 | 920.98 | 1157.77 | 1459.65 | 1271.80 | 1668.31 |
| The RMSL baselines are the average RMSL values for the eight regions over a 20-year period from 1986 to 2005. | | | | | | | |

Figure S9: Regional mean sea level forecast results in mm for the other six regions based on the ARDL models. (The curves for New York and Osaka are shown in the main text). Regional mean sea level forecast under no restriction scenario (red), COP26 restriction scenario (grey), SSP5-8.5 (orange), SSP3-7.0 (light-green), SSP4-6.0 (brown), SSP2-4.5 (dark-green), SSP1-2.6 (light-grey) and SSP1-1.9 (light-blue) from now till 2100; the uncertainties (forecast interval) under the corresponding scenario were shown by the red and grey shaded area. The forecast mean value, the 95% and the 99% forecast interval of 2100 and the mean value, the 99% forecast interval of 2050 were annotated by corresponding color from dark to light.

Table S8: Regional sea level forecasts for 2100. The column Avg. Diff (mm) shows the average difference between the yearly highest and mean sea levels. The columns HWL W/O R (mm) and HWL W R (mm) show the possible highest water level in 2100 under the without restriction and with restriction scenarios, respectively. They are calculated as the sum of the average difference between the yearly highest and mean sea levels^5^ and the 95 percent upper bound of the predicted yearly mean sea level for 2100. Using the 95 percent upper bound for forecast, the columns days W/O (day) and Percentage W/O (percent) show the number of days and percent of the time in 2100 that the regional sea level will rise more than 2 meters compared to 2021/2020 levels, and the columns days W (day) and Percentage W (percent) show these statistics under the restriction scenario.

| **Region** | **Avg. Diff (mm)** | **HWL W/O R (mm)** | **HWL W R (mm)** | **days W/O (day)** | **Percentage W/O (%)** | **days W (day)** | **Percentage W (%)** |
| --- | --- | --- | --- | --- | --- | --- | --- |
| New York | 1536.93 | 2662.64 | 2437.56 | 45.54 | 1.17% | 6.29 | 0.16% |
| Osaka | 1138.95 | 2326.49 | 2098.60 | 3.55 | 0.09% | 0.38 | 0.01% |
| Shanghai | 3576.10 | 4715.15 | 4483.11 | 345.27 | 28.71% | 319.58 | 23.09% |
| Hawaii | 700.76 | 1767.70 | 1561.80 | 0.01 | 0.00% | 0.00 | 0.00% |
| London | 2949.65 | 3590.27 | 3468.36 | 282.81 | 16.82% | 255.27 | 13.91% |
| Data were obtained from Japan Oceanographic Data Center (JODC) for Osaka^4^ and University of Hawaii Sea Level Center (UHSLC) for all the other locations^5^. The hourly data for New York, Shanghai, Hawaii, and London is from New York (the Battery) Station, Lusi Station, Hali Station, and Newlyn station, respectively. | | | | | | | |

## Permafrost Effect on Greenhouse Gas Emission

## According to Schuur and colleagues^6^, Arctic permafrost will, under a low global warming scenario (RCP 2.6), emit from the baseline of 0.332 Pg C of CO_2_ in 2021 to 0.449 Pg C of CO_2_ in 2099 in a linearly increasing pattern; and, from 0.344 Pg C to 1.436 Pg C or 0.630 Pg C to 2.970 Pg C, respectively, under a medium (RCP4.5 - RCP8.5) or a high (RCP 8.5) global warming scenario. Meanwhile, permafrost related CH_4_ emission will increase linearly from a baseline volume of 5 Tg C in 2021 to 21 Tg C in 2099 in a low global warming scenario, and, from 12 Tg C to 51 Tg C, or and 22 Tg C to 100 Tg C, respectively, under a medium or a high global warming scenario. As shown in the main text results section, the unrestricted scenario in our work corresponds largely to the medium global warming scenario while the COP26 scenario matches the low global warming scenario well. Therefore, the cumulative emission of CO_2_ and CH_4_ from the Arctic permafrost under the low or medium global warming scenario is added to the GWP forecast based on the COP26 scenario or the unrestricted scenario, respectively.

The equations of the annual emission of CO_2_ and additional CH­_4_ from arctic permafrost under different global warming scenario are as follows:

| Low global warming scenario: |  |
| --- | --- |
| CO_2_: | $0.332 + (year - 2021) * 0.0015$ (Pg C) |
| Additional CH_4_: | $5 + (year - 2021) * 0.2$ (Tg C) |
| Medium global warming scenario: |  |
| CO_2_: | $0.344 + (year - 2021) * 0.014$(Pg C) |
| Additional CH_4_: | $12 + (year - 2021) * 0.5$ (Tg C) |
| High global warming scenario: |  |
| CO_2_: | $0.63 + (year - 2021) * 0.03$ (Pg C) |
| Additional CH_4_: | $22 + (year - 2021) * 1$ (Tg C) |

## We adopted the conversion rate from atmosphere concentration to mass of carbon as:

##

$$\left\{ \begin{aligned} 1 ppm by volume of automosphere {CO}_{2}=2.13 Pg C \\ 1 ppb by volume of automosphere {CH}_{4}=2.06 Tg C \end{aligned} \right.$$


Figure S10: Global warming potential (GWP) projections with or without considering greenhouse gases emitted by permafrost.

## Data Availability

Data for all the Regional Mean Sea Level (RMSL) data are obtained from the Permanent Service for Mean Sea Level (PSMSL): <http://www.psmsl.org/data/obtaining/> (Retrieved on May 27th, 2022). Data containing New York, Shanghai, London, and Hawaii hourly water level are obtained from University of Hawaii Sea Level Center (UHSLC) <https://uhslc.soest.hawaii.edu/datainfo/> (Retrieved on Apr 29th, 2022). Osaka hourly data are obtained from Japan Oceanographic Data Center (JODC): <https://jdoss1.jodc.go.jp/vpage/tide.html> (Retrieved on Apr 29th, 2022).

## Reference

1. Sasaki, Y. N., Washizu, R., Yasuda, T. & Minobe, S. Sea level variability around Japan during the twentieth century simulated by a regional ocean model. *Journal of Climate* **30**, 5585–5595 (2017).

2. Permanent Service for Mean Sea Level (PSMSL). Tide Gauge Data, Retrieved 09 April 2022 from http://www.psmsl.org/data/obtaining/.

3. Holgate, S. J. *et al.* New data systems and products at the permanent service for mean sea level. *Journal of Coastal Research* **29**, 493–504 (2013).

4. Hydrographic Department: Maritime Safety Agency. Japan Oceanographic Data Center (JODC). (Brochure). (1987).

5. Caldwell, P., Merrifield, M. & Thompson, P. Sea level measured by tide gauges from global oceans–the Joint Archive for Sea Level holdings (NCEI Accession 0019568), Version 5.5, NOAA National Centers for Environmental Information, Dataset. *Centers Environ. Information, Dataset* **10**, V5V40S7W (2015).

6. Schuur, E. A. *et al.* Permafrost and climate change: carbon cycle feedbacks from the warming Arctic. *Annual Review of Environment and Resources* **47**, 343–371 (2022).
